# Standardization of criteria in MacCAT‐T and MacCAT‐CR for monoclonal anti–beta‐amyloid antibodies: A Delphi study

**DOI:** 10.1002/dad2.70112

**Published:** 2025-05-11

**Authors:** Jonas Karneboge, Ferdinand von Boehn, Julia Haberstroh

**Affiliations:** ^1^ Department of Psychology University of Siegen Siegen Germany

**Keywords:** anti‐amyloid therapy, capacity assessment, capacity to consent, cognitive impairment, dementia, informed consent, mental capacity

## Abstract

**Introduction:**

Assessing capacity to consent to treatment and participation in clinical research with monoclonal anti–beta‐amyloid antibodies is critical, especially given the frequent uncertainty in the eligible population. Capacity tends to be underestimated in Alzheimer's patients and overestimated in those with mild cognitive impairment (MCI).

**Methods:**

Using the Delphi method, an international expert panel (*N* = 21) was surveyed in two waves.

**Results:**

The participants reached consensus on 85 % of identified features, 90 % of benefits, and 88 % of risks.

**Discussion:**

The resulting standard emphasizes the understanding subscale of the MacArthur competence assessment tools (MacCAT) for both treatment and research, supporting use across clinical and research settings. Despite proven utility, only 4 % of psychiatrists currently use tools like MacArthur Competence Assessment Tool for Treatment (MacCAT‐T). This consensus aims to promote wider adoption of capacity assessments, integrating them routinely into clinical practice to balance patient autonomy with beneficence.

## 1 INTRODUCTION

The introduction of monoclonal anti‐beta amyloid antibodies as a disease‐modifying treatment for people with mild cognitive impairment (MCI) and early‐stage Alzheimer's disease (AD) poses new challenges for clinical prescribing practices.

These therapies—currently represented by the approved therapeutics lecanemab and donanemab in some countries—provide a first causal approach to the treatment of amyloid‐associated dementia, but are associated with specific risks, such as an increased incidence of amyloid‐related imaging abnormality (ARIA), including cerebral edema and microhemorrhages.^1^, ^2^ Given these risks, Karneboge et al.^3^ emphasize that patients must retain the capacity for autonomous treatment decisions, with clinicians ensuring they are fully informed and supported throughout the decision‐making process. This is particularly crucial as the complexity of the treatments and their potential side effects necessitate a decision‐making approach that respects patient autonomy while providing sufficient clinical guidance to navigate the risks and benefits effectively.

However, this patient group is at risk of having their capacity to consent prematurely questioned or underestimated by both family members^4^, ^5^ and healthcare providers,^6^, ^7^ starting at the time of diagnosis. Autonomous treatment decisions require the capacity to consent to treatment, but healthcare providers and family members often question or challenge this capacity based on the diagnosis alone, even in cases of MCI and early Alzheimer's disease, which is the target population for anti‐beta‐amyloid antibodies. Overestimating a person's capacity to consent neglects primarily the ethical principle of non‐maleficence according to Beauchamp and Childress' principles of biomedical ethics,^8^‐for example, when someone who does not fully understand the risk of ARIAs chooses a treatment and experiences severe side effects. Conversely, underestimating capacity violates the ethical principle of autonomy, for example, when a person is denied treatment because of presumed incapacity, even though they would rather accept the risks of ARIA than face disease progression untreated. This is particularly important because people with MCI are often assumed to have capacity due to the mild nature of their diagnosis,^9^, ^10^ although studies show a prevalence of incapacity of up to 20% of cases.^11^ This highlights the need for appropriate and valid assessments of capacity to consent to the prescription of antibody therapies.

Amaral et al.^12^ lists 17 standardized tools for assessing capacity to consent in medical contexts, of which the MacArthur Competence Assessment Tool for Treatment (MacCAT‐T) is recognized as the gold standard in the literature. While the MacCAT‐T is widely used in research as an outcome measure for decision‐making capacity interventions (distinct from the MacCAT‐CR's role in determining research eligibility), a recent survey with psychiatrists found that only 4% actually use it in clinical practice.^13^ Although the tool can be administered in approximately 20–25 min, the need for customization to fit each individual case presents a significant challenge for its use in clinical practice. In contrast, clinical judgment without standardized tools is often unreliable, as noted by Marson et al.^14^ and Haberstroh & Mueller.^15^ The MacCAT‐T and its counterpart, the MacArthur Competence Assessment Tool for Clinical Research (MacCAT‐CR), assess four key abilities related to decision‐making capacity: Understanding, appreciation, reasoning, and expressing a choice.^16^ Understanding refers to the ability to comprehend treatment‐relevant information including the nature of one's condition, treatment options, and associated risks and benefits. Appreciation involves recognizing how the disclosed information applies to one's own situation and acknowledging the potential consequences. Reasoning encompasses the ability to engage with treatment‐related information in a rational way, comparing options and their consequences. Expressing a choice requires the ability to communicate a clear and consistent treatment decision. For clinical research (MacCAT‐CR), these components are similarly structured but focus on research participation rather than treatment. Of these, *understanding* is the most context‐dependent and thus requires adaptation based on the specific treatment or research protocol in question.^17^

Although these criteria are not expected to provide complete information,^17^ selecting the right ones is crucial to cover the full spectrum of treatment and disease‐related benefits and risks. According to the MacCAT‐T and MacCAT‐CR manuals, clinicians should focus on the two or three most important aspects of information for each domain (features, benefits, risks). Given the importance of autonomous decision‐making in the context of antibody therapies—and the potential lack of capacity to consent in patients with early AD or MCI—a valid and reliable assessment is critical, especially when weighing the risks of ARIA. In this context, the balance between the ethical principles of autonomy and beneficence becomes a central concern.

This Delphi study aims to establish expert‐consented criteria to standardize the assessment of the *understanding* component of the MacCAT‐T and MacCAT‐CR by consensus, specifically for use in decisions related to monoclonal anti‐beta‐amyloid antibodies. This will ensure that the tools can provide valid, practical, and standardized assessments for critical treatment decisions, such as whether or not to accfept the risks associated with antibody therapies.

## 2 METHODS

The consensus Delphi^18^ aimed to agree on the treatment characteristics, benefits, and risks of lecanemab and donanemab. For this purpose, this information was extracted from the prescribing information for the drugs Lequembi (active ingredient: lecanemab)^19^ and Kinsula (active ingredient: donanemab)^20^ and from the Appropriate Use Recommendations for Lecanemab.^21^

We aimed for a sample size of at least 18 experts^22^ to strike an effective balance between expertise and diversity of opinion.^23^

### 2.1 Consensus group

Invitations for the first wave of the Delphi survey were sent to experienced clinicians from a German professional network of memory clinics who routinely assess cognitive and functional capacity in people with dementia, as well as to selected international experts with established expertise in behavioral neurology, neuroimaging, geriatrics, and clinical assessment methods for different types of dementia. In total, 120 potential respondents were contacted. A total of *N*_1_ = 25 participants from Germany, Finland, Switzerland, Belgium, and France participated in the first wave. By discipline, 15 (60.0 %) identified with neurology, 7 (28.0 %) with psychiatry, 5 (20.0 %) with neuropsychology, 2 (8.0 %) with psychotherapy, and 5 (20.0 %) with other disciplines (geriatrics, medical ethics, and palliative care)—multiple answers were allowed. A total of *n* = 22 (88.0 %) reported that they assess patients’ capacity to consent as part of their professional practices, with the majority (81.82 %) doing so at least several times a month. The most common frequency was *several times a month* (40.91 %).

In the second wave of the Delphi survey, *N*_2_ = 21 participants attended. Only participants of the first wave had access to the second wave of the Delphi survey.

### 2.2 Materials

The materials used in this study included the full prescribing information for lecanemab and donanemab, as well as the Appropriate Use Recommendations for Lecanemab.^21^ These documents were analyzed to extract treatment *features*, *benefits*, and *risks*, which were then presented to the experts for review. In total, nine features, six benefits, and 21 risks were identified a priori for lecanemab, and 10 features, five benefits, and 16 risks for donanemab (see Tables 1, 2, 3).

**TABLE 1:** Delphi consensus on the features of treatment

Lecanemab	W1	W2	Donanemab	W1	W2
Ongoing treatment	40.0%	0.0%	Limited‐duration treatment regimen based on amyloid plaque removal	32.0%	0.0%
Every 2 weeks	64.0%	100.0%	Amyloid PET values may increase after treatment	0.0%	0.0%
Intravenous infusion	64.0%	90.0%	Every 4 weeks	72.0%	95.2%
Each infusion takes about 1 h	8.0%	0.0%	Intravenous infusion	76.0%	95.2%
30 min of post‐infusion monitoring (2–3 h for the first three infusions)	0.0%	0.0%	Each infusion lasts about 30 min	8.0%	0.0%
Outpatient treatment requires travel for appointments	20.0%	0.0%	30 min of post‐infusion monitoring	0.0%	0.0%
Three to five brain MRIs in the first year	60.0%	95.0%	Outpatient treatment requires travel for appointments	20.0%	0.0%
Suspension or termination of treatment for side effects	24.0%	0.0%	Four to five brain MRIs in the first year	52.0%	90.5%
Monoclonal antibody against beta‐amyloid plaques	8.0%	0.0%	Suspension or termination of treatment for side effects	12.0%	0.0%
			Monoclonal antibody against beta‐amyloid plaques	8.0%	0.0%

Abbreviations: MRI, magnetic resonance imaging; PET, positron emission tomography; W1, Delphi Wave 1; W2, Delphi Wave 2.

**TABLE 2:** Delphi consensus on the benefits of treatment.

Lecanemab	W1	W2	Donanemab	W1	W2
25%–47% reduction in cognitive decline compared to PLC	80.0%	100.0%	20–29% reduction in cognitive decline compared to PLC	80.0%	95.2%
37% reduction in functional decline compared to PLC	88.0%	95.2%	28% reduction in functional decline compared to PLC	92.0%	95.2%
59%–61% reduction in amyloid beta PET Centiloid plaques	32.0%	0.0%	83% reduction in amyloid beta PET Centiloid plaques	28.0%	0.0%
22% reduction in amyloid beta PET Composite SUVR	0.0%	0.0%	31% reduction in amyloid beta PET composite SUVR	0.0%	0.0%
16%–24% reduction in plasma p‐tau181	0.0%	0.0%	33% reduction in plasma p‐tau217	0.0%	0.0%
9% increase in plasma Aβ 42/40 ratio	0.0%	0.0%			

Abbreviations: PET, positron emission tomography; PLC, placebo; SUVR, standardized uptake value ratio; W1, Delphi Wave 1; W2, Delphi Wave 2.

In addition, extracts from the (MacCAT‐T)^17^ and (MacCAT‐CR)^24^ manuals were used to guide participants in the selection of relevant items. Specifically, participants were instructed to focus on the *understanding* component, which is the most context‐specific, and to identify the most relevant features, benefits, and risks of the treatment according to the guidelines outlined in these manuals.

### 2.3 Delphi process

A two‐wave Delphi consensus approach was employed in this study, as outlined by.^18^ In this process, experts were guided through a structured decision‐making framework aimed at establishing consensus on the most important treatment characteristics, benefits, and risks of lecanemab and donanemab.

At the beginning of the Delphi procedure, participants were given detailed instructions on the structure of the MacCAT‐T and MacCAT‐CR, with emphasis on the selective assessment of the *understanding* part, as noted by.^17^ The experts were informed that the aim of the tools was not to replace patient disclosure but to evaluate the most critical elements that contribute to the patient's *understanding* of treatment decisions.

In the first wave of the Delphi survey, experts were presented with information extracted from the prescribing information for lecanemab and donanemab. These data included *features*, *benefits*, and *risks* of the treatments. Experts were also able to review the full prescribing information if needed, ensuring they could make informed selections. The experts were instructed to select the three most important *features*, the two most important *benefits*, and the two most important *risks*, which would serve as the basis for assessing the understanding component of capacity to consent.

In the second wave, the same experts were presented with the results of the first wave. They were asked to review these results and make a new selection of key *features*, *benefits*, and *risks* based on the collective ratings of the first wave, refining their choices to reach a consensus.

## 3 RESULTS

### 3.1 Delphi outputs

In the first wave, there was already a clear trend among the experts, so that for lecanemab 5/9 in the subpart of features, 3/6 in the subpart of benefits, and 17/21 in the area of risks received less than 25.0 % agreement, and for donanemab 6/10, 2/5, and 13/16, respectively. In the second wave, consensus was reached for lecanemab on three features with an agreement ranging from 90.0 % to 100.0 %, two benefits with an agreement ranging from 95.2 % to 100.0 %, and two risks, with an agreement ranging from 80.9 % to 90.5 %. For donanemab consensus could be reached on three features with an agreement ranging from 90.5 % to 95.2 %, two benefits with an agreement of 95.2 %, and two risks with an agreement ranging from 80.9 % to 100.0 %; see Tables 1, 2, 3.

**TABLE 3:** Delphi consensus on the risks of treatment 1/2 and 2/2.

Lecanemab	W1	W2	Donanemab	W1	W2
ARIA (21% compared to 9% PLC)	44.0%	28.6%	ARIA (36% compared to 14% PLC)	48.0%	0.0%
3% symptomatic ARIA	20.0%	80.9%	6% symptomatic ARIA	52.0%	80.9%
Symptomatic ARIA with increased risk of headache, confusion, visual changes, dizziness, nausea, and gait difficulty	40.0%	0.0%	ARIA related symptoms resolve for 85% of patients	12.0%	0.0%
100% of ARIA incidents resolve	0.0%	0.0%	Increased ARIA risk in ApoE ε4 homozygotes and heterozygotes	8.0%	0.0%
Increased ARIA risk in ApoE ε4 homozygotes and heterozygotes	12.0%	0.0%	ARIA‐E (24% compared to 2% PLC)	8.0%	0.0%
ARIA‐E (10%–13% compared to 1%–2% PLC)	8.0%	0.0%	Headache (13% compared to 10% PLC)	4.0%	0.0%
Symptomatic ARIA‐E (3% compared to 0% PLC)	0.0%	0.0%	ARIA‐H (31% compared to 13% PLC)	4.0%	0.0%
Headache (11%–14% compared to 8%–0% PLC)	0.0%	0.0%	ARIA‐H microhemorrhage (25% compared to 11% PLC)	0.0%	0.0%
ARIA‐H (17% compared to 9% PLC)	8.0%	0.0%	ARIA‐H superficial siderosis (15% compared to 3% PLC)	4.0%	0.0%
Symptomatic ARIA‐H (0.7% compared to 0.2% PLC)	4.0%	0.0%	Severe intracerebral hemorrhage (0.5% compared to 0.2% PLC)	8.0%	0.0%
ARIA‐H microhemorrhage (14% compared to 8% PLC)	4.0%	0.0%	Infusion reactions (9% compared to 0.5% placebo) including chills, erythema, nausea/vomiting, difficulty breathing/dyspnea, sweating, elevated blood pressure, headache, chest pain, and low blood pressure	52.0%	100.0%
ARIA‐H superficial siderosis (6% compared to 3% PLC)	0.0%	0.0%	Intestinal obstruction (0.4% compared to 0% PLC)	0.0%	0.0%
Severe intracerebral hemorrhage (0.7% compared to 0.1% PLC)	12.0%	0.0%	Intestinal perforation (0.2% compared to 0.1% PLC)	0.0%	0.0%
Infusion reactions (20%–26% compared to 3%–7% placebo) including fever and flu‐like symptoms, nausea, vomiting, hypotension, hypertension, and oxygen desaturation	48.0%	90.5%	Hypersensitivity reactions (3% compared to 1% PLC)	0.0%	0.0%
Diarrhea (8% compared to 5% PLC)	0.0%	0.0%	87% Developing ADA with higher incidence of infusion‐related reactions compared to PLC	0.0%	0.0%
Nausea/vomiting (6% compared to 4% PLC)	0.0%	0.0%	Increased risk of death (0.1% compared to 0% with PLC)	0.0%	0.0%
Rash (6% compared to 4% PLC)	0.0%	0.0%			
Cough (9% compared to 5% PLC)	0.0%	0.0%			
Atrial fibrillation (3% compared to 2% PLC)	0.0%	0.0%			
41% Developing ADA	0.0%	0.0%			
Decreased lymphocyte count after first infusion (38% compared to 2% PLC)	0.0%	0.0%			

Abbreviations: ADA, anti‐drug antibodies; ApoE, apolipoprotein E; ARIA, amyloid‐related imaging abnormality; ARIA‐E, amyloid‐related imaging abnormality – edema; ARIA‐H, amyloid‐related imaging abnormality ‐ hemorrhage; PLC, placebo; W1, Delphi Wave 1; W2, Delphi Wave 2.

Among the features of lecanemab discussed by the experts in the second Delphi wave, the mode of administration was often highlighted, specifically “intravenous infusion” and “intravenous infusion in the clinic”. This was seen as a notable feature of the treatment. Another suggestion was to possibly expand on the standard statement of “three to five brain MRIs in the first year” of treatment by adding that additional MRIs may be required if side effects occur. However, this is not a feature of the drug itself but could be considered a risk, reflecting the need for careful monitoring in the event of adverse events.

Similarly, for donanemab, the experts highlighted the method of administration, with “infusion via vein” and “intravenous infusion in the clinic” being essential aspects of the treatment. This requirement for intravenous administration is a key feature, although it shares the same logistical concerns as lecanemab regarding the need for regular clinical visits, which could be a limiting factor for patient convenience.

In terms of benefits, feedback on both lecanemab and donanemab highlighted the importance of providing clear and realistic information about potential outcomes. One expert commented that for donanemab “it should be added what a 28% reduction means in absolute terms and that the clinical relevance is unclear”. Similarly, another expert commented on lecanemab: “It should be explained what the 37% reduction actually means”. These responses indicate a common concern among experts to ensure that patients receive transparent and unbiased information about the efficacy of these treatments, to help them form a realistic understanding of the potential benefits. Lastly, the experts did not contribute any discussion points on the risks.

### 3.2 Consensus

Based on the ratings from the two Delphi waves and the wording suggestions, the items presented in Table 4 were derived for the understanding component of the MacCAT‐T and MacCAT‐CR. An agreement of 80.9 %–100.0 % was reached across all items.

**TABLE 4:** Consented items for *understanding*‐subpart

Lecanemab	Donanemab
Every 2 weeks	Every 4 weeks
Intravenous infusion in the clinic	Intravenous infusion in the clinic
Three to five brain MRIs in the first year	Four to five brain MRIs in the first year
25%–47% reduction in cognitive decline compared to PLC	20%–29% reduction in cognitive decline compared to PLC
37% reduction in functional decline compared to PLC	28% reduction in functional decline compared to PLC
3% Symptomatic ARIA with increased risk of headache, confusion, visual changes, dizziness, nausea, and gait difficulty	6% symptomatic ARIA with increased risk of headache, confusion, visual changes, dizziness, nausea, and gait difficulty
Infusion reactions (20%–26% compared to 3%–7% placebo) including fever and flu‐like symptoms, nausea, vomiting, hypotension, hypertension, and oxygen desaturation	Infusion reactions (9% compared to 0.5% placebo) including chills, erythema, nausea/vomiting, difficulty breathing/dyspnea, sweating, elevated blood pressure, headache, chest pain, and low blood pressure

Abbreviations: ARIA, amyloid‐related imaging abnormality; MRI, magnetic resonance imaging; PLC, placebo.

The consensus items presented in Table 4 achieved agreement levels between 80.9% and 100%, representing strong consensus according to established Delphi methodology, where the agreement of 70% is typically considered general consensus and 80% strong consensus.^25^, ^26^ It should be emphasized that these standardized criteria serve as anchors for assessment and presuppose comprehensive disclosure about each item. For example, while the assessment focuses on understanding of “6% symptomatic ARIA” for donanemab, this assumes a prior thorough explanation of what ARIAs are, their symptoms, and their typical progression and resolution patterns. The MacCAT instruments are not designed to replace thorough patient disclosure but rather to assess whether key information from that disclosure has been understood and retained.

## 4 DISCUSSION

The aim of this study was to establish a consensus standard for assessing the capacity to consent to treatment with lecanemab and donanemab, two emerging antibody therapies for Alzheimer's disease. By engaging a panel of international experts using the Delphi method, we were able to identify key features, benefits, and risks that are essential for assessing patient understanding, a critical component of capacity to consent. This standard is intended to facilitate widespread use in both clinical practice, using the MacCAT‐T, and research settings, employing MacCAT‐CR. By providing a consistent and structured approach, we aim to help clinicians and researchers guide their assessments of patient autonomy. The initial low agreement rates in Wave 1, followed by strong consensus in Wave 2, reflect the diversity within our multidisciplinary expert panel and the complexity of these novel therapies. This variation in expert prioritization underscores the need for standardized assessment criteria, as without such guidance, capacity assessments may focus on different aspects of these treatments in different clinical settings. By creating a framework that is adaptable across various clinical and research contexts, we intend to support more informed and independent decision‐making by patients regarding whether to proceed with or refuse treatment with these antibody therapies. While this standardization fully applies to treatment‐related assessments using MacCAT‐T, for research settings using MacCAT‐CR it specifically addresses understanding items related to the medications themselves (such as administration, effects, and monitoring). Additional protocol‐specific understanding items in research settings must still be individually tailored to each study's unique procedures and design as specified in the MacCAT‐CR manual.

The experts in our study highlighted several critical aspects that need to be included in any assessment of capacity to consent. Features related to the mode of administration, such as intravenous infusion and the need for regular clinical visits, were seen as central to understanding the implications of these therapies. The importance of clarifying the real‐world impact of reported benefits, such as the 37% reduction in functional decline for lecanemab and the 28% reduction for donanemab, was also highlighted. Experts warned that without clear communication, these figures could be misleading, underscoring the need for a transparent explanation of treatment outcomes. In addition, the potential risks, particularly related to neurological side effects such as brain edema and bleeding, were identified as significant concerns that need to be fully understood by patients before making a decision.

**FIGURE 1:**
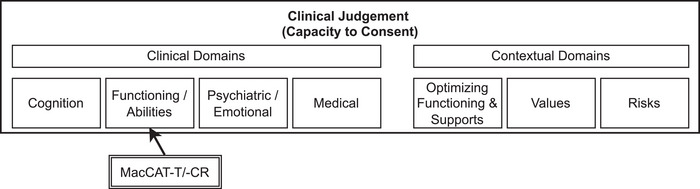
MacCAT‐T and MacCAT‐CR in context of clinical judgement. Adapted from American Bar Association/American Psychological Association.^27^ MacCAT‐CR, MacArthur Competence Assessment Tool for Clinical Research; MacCAT‐T, MacArthur Competence Assessment Tool for Treatment

Despite the value of this consensus, it is important to emphasize that this standard for assessing capacity should not be used in isolation. Capacity assessments are inherently complex and multifaceted, and while our findings provide a structured approach, they are only one component of a broader clinical judgment,^27^, ^28^ see Figure 1. Clinicians must continue to integrate these assessments with their overall understanding of the patient's condition, cognitive abilities, and preferences to ensure a holistic assessment that respects the patient's autonomy while safeguarding their interests. Moreover, as we did not assess our experts' prior experience with the MacCAT instruments, we cannot determine how their familiarity with these specific tools might have influenced their selection of assessment items and their understanding of how standardized criteria integrate into the overall assessment process.

While this study was primarily designed to standardize the assessment of capacity to consent via the MacCAT instruments, our findings have additional implications that deserve acknowledgment. The consensus items identified by our expert panel not only represent critical elements for capacity assessment but also highlight information that experts consider most essential for patients to understand when considering treatment with anti–beta‐amyloid antibodies.

However, it is crucial to emphasize that these consensus items should not be viewed as a replacement for comprehensive disclosure. Rather than encouraging abbreviated disclosure, these findings can serve as a quality assurance framework to ensure that, at minimum, these key elements are thoroughly addressed during the informed consent process. In clinical practice, where the actual disclosure (actual) may sometimes fall short of the required standard (target), our results provide practitioners with guidance on the essential content that must be conveyed and understood. This may be particularly valuable in busy clinical settings where time constraints are a reality.

By identifying the most critical information for understanding these novel therapies, our findings can help bridge the gap between the theoretical ideal of complete disclosure and the practical realities of clinical practice, ultimately serving to strengthen patient autonomy in treatment decisions. These standardized criteria can thus function as both assessment anchors and as quality indicators for the disclosure process itself, ensuring that critical information is not overlooked in discussions with patients.

Furthermore, while the MacCAT‐T and MacCAT‐CR remain a widely accepted tool for assessing capacity, they are not without limitations. The MacCAT‐T is strongly dependent on the verbal abilities of the patients, especially with regard to verbal recall, which might put people with dementia at a disadvantage, so that the use of additional tools for decisional support may be required during application.^15^ The MacCAT‐CR already addresses this challenge by providing disclosure cards for each section as part of its standard procedure.^24^ Likewise, as highlighted by Gilbert et al.^29^ the use of the MacCAT‐T–which is equally applicable to the MacCAT‐CR–can be time‐consuming and often requires considerable effort to administer in busy clinical and research settings. This may be a barrier to its widespread use in practice, particularly in contexts where time and resources are limited. Therefore, while the consensus criteria derived from our study provide a valuable guide, there is still a need for practical solutions that balance thoroughness with feasibility in real‐world settings.

In conclusion, this study represents a step toward improving the assessment of capacity to consent in the context of antibody therapies and aims to encourage more widespread adoption of capacity assessments and their routine integration into clinical practice. By incorporating expert opinion, we have created a framework that aims to strike a balance between respecting patient autonomy and upholding the principle of beneficence. This approach might help clinicians and researchers facilitate informed, autonomous decision‐making while also ensuring that patients’ well‐being is prioritized. A standardized and routinely conducted assessment is especially important for patients with MCI, as they are often assumed to be capable of consenting despite a 20 % prevalence of incapacity. This highlights the critical need for reliable tools to ensure that treatment decisions are made autonomously and ethically reasonable. However, as with any assessment tool, it is important that these standards are applied within the broader context of clinical judgment and that they contribute to, rather than replace, a comprehensive evaluation of a patient's capacity to consent.

## CONFLICT OF INTEREST STATEMENT

The authors declare no conflicts of interest. Author disclosures are available in the Supporting Information.

## ETHICS STATEMENT

The Ethics Committee of University Siegen, Germany, reviewed and approved the study on August 7, 2024 (LS_ER_30_2024). All participants gave written informed consent prior to participation. Information was provided on the purpose of the study, procedures, data handling, expected results, and contact details. Participants were informed that participation is voluntary and that they may withdraw at any time without explanation.

## CONSENT STATEMENT

All participants in this study provided written informed consent prior to participation. They were informed about the purpose of the study, the procedures involved, data handling, expected outcomes, and contact details for further inquiries. Participation was entirely voluntary, and participants were made aware that they could withdraw at any time without any repercussions. This study was reviewed and approved by the Ethics Committee of the University of Siegen (Approval No. LS_ER_30_2024).

## Supporting information



Supporting Information
